# Digital Health Competence and Attitudes Toward AI Among Health Care Professionals: Convergent Mixed Methods Study

**DOI:** 10.2196/96170

**Published:** 2026-08-24

**Authors:** Segundo Jimenez-Garcia, Manuela Domingo-Pozo, Jose Garcia-Rodriguez, David Tomás, David Ortiz-Perez, Kristina Mikkonen, Erika Jarva, M Flores Vizcaya-Moreno

**Affiliations:** 1 Department of Nursing, Faculty of Health Sciences, University of Alicante, Carretera San Vicente del Raspeig s/n, Alicante, Alicante, 03690, Spain, 34 965 903 400 ext 2120; 2 Institute for Health and Biomedical Research of Alicante (ISABIAL), Alicante, Alicante, Spain; 3 Department of Computer Science and Technology, Polytechnic School, University of Alicante, Alicante, Alicante, Spain; 4 Department of Software and Computing Systems, Polytechnic School, University of Alicante, Alicante, Alicante, Spain; 5 Research Unit of Health Sciences and Technology, Faculty of Medicine, University of Oulu, Oulu, North Ostrobothnia, Finland; 6 Medical Research Centre, Oulu University Hospital, Oulu, North Ostrobothnia, Finland; 7 Department of Nursing, Midwifery and Health, Faculty of Health and Life Sciences, Northumbria University, Newcastle, England, United Kingdom; 8 Department of Evidence-Based Clinical Nursing, Division of Health Sciences, Graduate School of Medicine, The University of Osaka, Suita, Osaka, Japan

**Keywords:** AI, digital health competence, digital health, digital technology, health personnel, professional competence, mixed-methods research.

## Abstract

**Background:**

Digital transformation is reshaping health care systems and requires health care professionals to develop advanced digital health competencies. The integration of AI into clinical practice introduces new demands related to critical evaluation, human oversight, ethical responsibility, and regulatory compliance. However, evidence linking validated measures of digital health competence with health care professionals’ attitudes toward AI remains limited.

**Objective:**

This study aimed to (1) assess digital health competence among health care professionals in Spain, (2) examine organizational conditions supporting competence development, (3) explore perceptions about AI use in the workplace, and (4) examine the association between digital health competence and attitudes toward AI.

**Methods:**

A national cross-sectional convergent mixed methods study was conducted between November 2023 and January 2024 with a voluntary convenience sample of 229 health care professionals. Digital health competence was assessed using DigiHealthCom (digital health competence instrument; 42 items, 5 domains) and DigiComInf (the aspects associated with digital health competence instrument; 15 items, 3 domains). Confirmatory factor analysis evaluated structural validity. Open-ended responses regarding AI perceptions were explored using inductive qualitative content analysis. AI attitudes were classified into 4 categories (positive, negative, ambivalent, and uncertain), and their association with digital health competence was examined using multinomial logistic regression adjusted for age and sex.

**Results:**

Participants were predominantly female (169/229, 73.8%) and nurses (123/229, 53.7%), with a mean age of 45.9 (SD 10) years. Overall digital health competence was moderate (mean 3.01, SD 0.51), with the highest scores in information and communication technology competence (3.34, SD 0.63) and the lowest in competence related to evaluating and implementing digital solutions (2.83, SD 0.63). Organizational and educational support for competence development was also moderate (mean 2.51, SD 0.57), with organizational planning receiving the lowest DigiComInf scores (2.21, SD 0.76). Confirmatory factor analysis supported the proposed factor structures for both instruments (DigiHealthCom: comparative fit index=0.95, root-mean-square error of approximation=0.049; DigiComInf: comparative fit index=0.95, root-mean-square error of approximation=0.084). Most participants expressed positive attitudes toward AI in the workplace (134/228, 58.77%). Qualitative findings revealed a pattern of conditional optimism, with expected benefits for efficiency and patient care balanced by concerns regarding training, governance, regulation, and human oversight.

**Conclusions:**

Digital health competence was positively associated with health care professionals’ attitudes toward AI. However, the cross-sectional design does not allow conclusions regarding the direction of this association. Deficiencies in higher-order competencies in evaluation and implementation, together with limited organizational support, highlight areas that may benefit from targeted educational and organizational strategies to promote the safe and responsible use of AI in health care.

## Introduction

Digital transformation has become a structural priority for health systems worldwide. The World Health Organization’s Global Strategy on Digital Health 2020‐2025 defines digital health interventions as the use of digital technologies to strengthen health system performance and patient outcomes [1]. Within this framework, digital health competence refers to the integrated set of knowledge, skills, and attitudes enabling health care professionals to use digital technologies safely, effectively, and ethically in the workplace.

Early work defined digital competence as informatics proficiency [2]. Modern frameworks stress broader, multidimensional skills, including telehealth communication, data literacy, critical digital tools appraisal, and ethical awareness [3-6]. Despite increasing conceptual refinement, systematic reviews continue to report heterogeneity in definitions and a limited number of psychometrically validated instruments tailored specifically to health care professionals, while also highlighting that competence extends beyond technical skills to include evaluative and patient-centered dimensions [7,8].

The rapid integration of AI into health care has introduced a related but conceptually distinct construct: AI competence or AI literacy. While digital health competence encompasses general digital capabilities, AI competence involves the ability to understand, critically evaluate, supervise, and responsibly use AI-driven systems in clinical contexts [9]. This includes knowledge of algorithmic bias, explainability, accountability, regulatory frameworks, and professional liability. The distinction is increasingly relevant as AI applications, ranging from clinical decision support to generative documentation systems, are embedded in routine care pathways [10].

Recent empirical studies indicate that AI-supported tools are becoming increasingly integrated into the daily work of health care professionals. Studies conducted in clinical settings report that health care professionals generally view AI-assisted decision support positively and perceive it as having the potential to improve diagnostic accuracy and efficiency [11]. However, acceptance is conditional. Likewise, physician surveys report cautious acceptance of AI-assisted diagnostic tools, contingent on integration into clinical workflows, reliability, and clarity regarding clinical responsibility [12,13].

Across professional groups, concerns persist regarding data protection, bias, erosion of professional autonomy, and potential depersonalization of care [14]. Qualitative studies further indicate that expectations of improved efficiency coexist with concerns about insufficient training and the lack of clear ethical and regulatory frameworks for AI implementation [15]. The literature concludes that usefulness must be balanced with ethical, organizational, and professional concerns.

From a theoretical perspective, technology adoption frameworks such as the technology acceptance model (TAM) and the unified theory of acceptance and use of technology (UTAUT) provide a conceptual basis for understanding health care professionals’ engagement with digital technologies, emphasizing the role of perceived usefulness, ease of use, social influence, facilitating conditions, and trust in shaping intention to use AI-enabled systems [16-19]. These frameworks explain technology acceptance but do not assess digital health competence itself. In this study, they were therefore used to inform the theoretical interpretation of the relationship between digital health competence and attitudes toward AI rather than as measurement models. Nonetheless, empirical studies explicitly linking validated measures of digital health competence with real-world professional attitudes toward AI remain scarce, despite the growing availability of psychometrically validated competency instruments and ongoing concerns about measurement and reporting gaps [20,21]. Little is known about how baseline digital competence relates to clinicians’ perceptions of AI within specific national health care systems, as existing national-level evidence often focuses on trust, acceptance, or implementation experiences rather than directly modeling competence-attitude pathways [22,23]. Addressing this gap requires moving beyond descriptive competence profiling toward examining the association between digital health competence and attitudes toward AI.

To address measurement gaps, Jarva et al [24] developed the DigiHealthCom (digital health competence instrument) and DigiComInf (aspects associated with digital health competence instrument) to assess digital health competence and its associated factors. Subsequent cross-cultural validation studies [21,25,26] and large international analyses [27] have supported the model’s structural validity and underscored the importance of managerial and collegial support in competence development. Although previous research using DigiHealthCom and DigiComInf has frequently relied on cluster-based segmentation to identify competence profiles, fewer studies have examined digital health competence as a continuous predictor of AI-related attitudes within theory-informed analytical frameworks.

Despite accelerating digital transformation and increasing AI integration across European health care systems, empirical research examining Spanish health care professionals’ digital health competence and attitudes toward AI in the workplace remains limited. This gap is particularly salient given the increasing use of AI-driven technologies in the Spanish National Health System [28] and the evolving regulatory environment under the EU (European Union) Artificial Intelligence Act [29]. Understanding how competence levels relate to professional attitudes is essential to inform targeted training strategies, organizational policies, and the responsible implementation of AI in clinical settings.

This study aims to (1) describe digital health competence among health care professionals in Spain, (2) characterize organizational and educational conditions supporting digital competence development, (3) explore expected impacts of AI on professional practice, and (4) examine the association between digital health competence and AI attitudes, including themes derived from open-ended responses. Based on technology adoption theory (TAM/UTAUT), we hypothesized that higher digital health competence would be associated with a greater likelihood of expressing a positive attitude toward AI in the workplace.

## Methods

### Research Design

**Figure 1.**
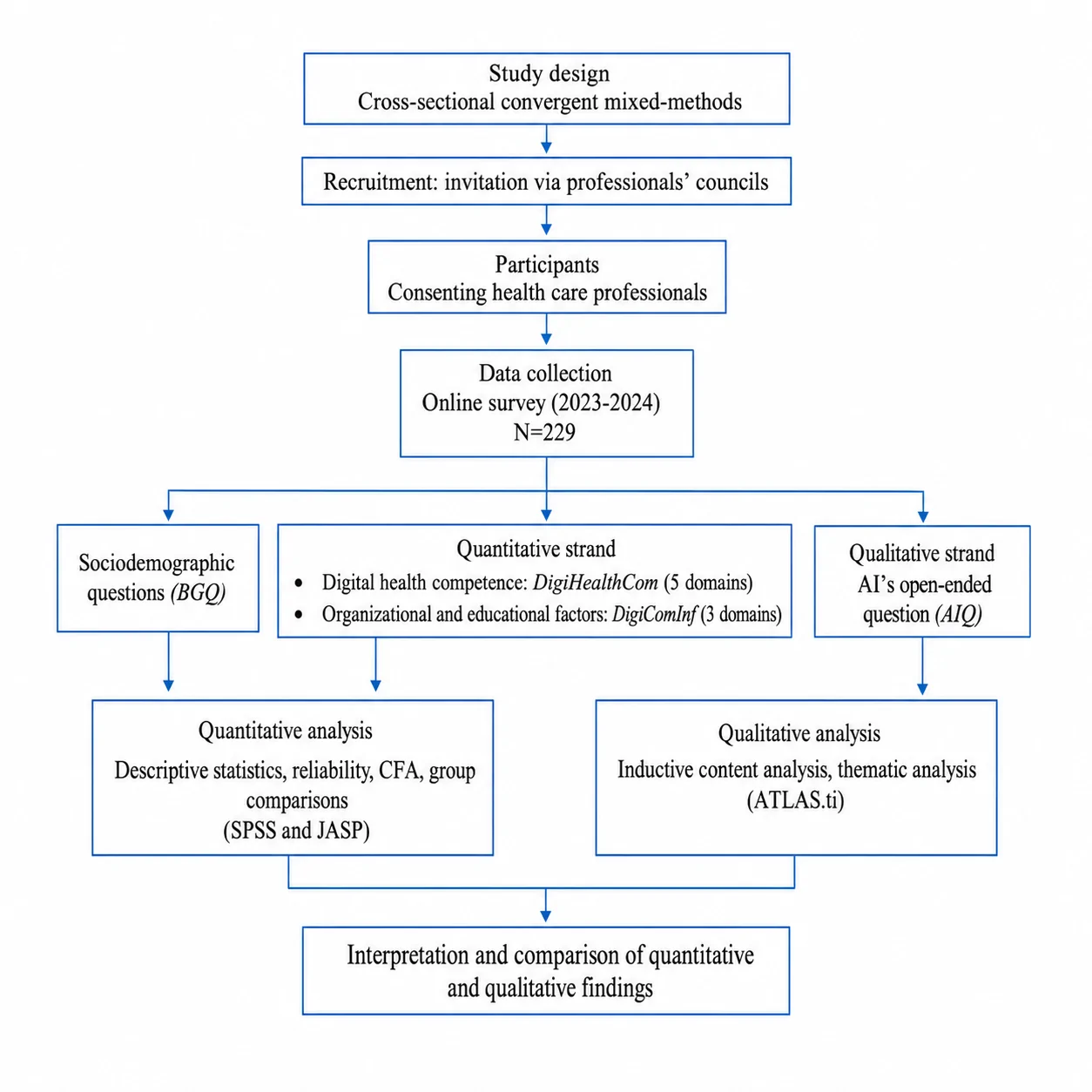
Study design and convergent mixed methods workflow. AIQ: AI question; BGQ: background question; CFA: confirmatory factor analysis.

A national cross-sectional convergent mixed methods study was conducted using a self-administered electronic survey from November 2023 to January 2024 (Figure 1). Quantitative and qualitative data were collected concurrently and analyzed in parallel. This study followed the STROBE (Strengthening the Reporting of Observational Studies in Epidemiology) guidelines to ensure transparency and rigor in reporting [30] (Checklist 1).

The research objectives (RO) were explicitly aligned with the selected instruments and data sources to ensure conceptual and methodological coherence. Perceived digital health competence (RO1) was operationalized using the DigiHealthCom, which captures multiple dimensions of health care professionals’ digital health competence. Organizational and educational factors that facilitate or hinder competence development (RO2) were assessed using the DigiComInf, focusing on managerial support, organizational practices, and collegial influence. Perceptions of AI in professional practice (RO3) were explored qualitatively through an open-ended question, allowing participants to articulate expectations, concerns, and perceived implications of AI without predefined response constraints. Finally, to examine the association between digital health competence and AI attitude (RO4), AI question (AIQ) responses were quantified into 4 mutually exclusive attitude categories and, using Participant Identification Code, linked to DigiHealthCom for inferential testing using multinomial logistic regression, adjusted for age and sex.

Integration was conducted at the interpretation stage. Quantitative findings on competence levels were compared with qualitative themes regarding competence development, training needs, and governance concerns to identify convergence, divergence, and complementarity. The quantitated AI attitude variable enabled direct modeling of competence-attitude associations.

### Target Population and Participants

A voluntary convenience sampling strategy through professional bodies was used [31]. The target population was invited through national professional councils via institutional mailing lists and public dissemination channels. Participation was voluntary, and all eligible professionals who accessed the survey during the data collection period were included. The exact response rate could not be calculated because the survey was disseminated through multiple professional mailing lists.

### Instruments

In this study, the data were collected using an online survey structured into 3 parts. The first part included background questions that described this study’s sample (sociodemographic data, professional characteristics, and digital use).

The second part comprised the DigiHealthCom and DigiComInf [24]. The final part consisted of an open-ended question designed to explore participants’ perceptions of the use of AI in the workplace and its potential applications in professional contexts (the AIQ).

The DigiHealthCom comprises 5 domains (42 items) and is used to assess health care professionals’ digital health competence. It covers the following domains: human-centered remote counseling competence (16 items), digital solutions as part of work (9 items), information and communication technology (ICT) competence (5 items), competence in using and evaluating digital solutions (8 items), and ethical competence related to digital solutions (4 items). The DigiComInf comprises 3 domains (15 items) that measure educational and organizational factors associated with digital health competence, including support from management (6 items), organizational practices for digital competence development (4 items), and colleagues’ adoption and influence (5 items). Both instruments used a 4-point Likert scale for scoring (1=completely disagree, 2=partially disagree, 3=partially agree, 4=completely agree). Both instruments were originally developed and psychometrically validated in the Finnish population, demonstrating satisfactory face and content validity and reliability [24]. DigiHealthCom and DigiComInf were translated from English into Spanish for the international study by Mikkonen et al [27]. An independent back-translation into English was subsequently performed by 2 researchers, and the translated versions were compared with the original instruments to ensure conceptual and semantic equivalence.

The content validity of the final survey, including DigiHealthCom, DigiComInf, the AIQ, and the background questions, was evaluated by a panel of 10 national experts to ensure the instruments’ relevance and applicability in the Spanish context. Content validity was assessed using the content validity index, applying both the item-level method (I-CVI) and the scale-level averaging method (S-CVI/Ave) [32].

### Data Collection

Data were collected between November 2023 and January 2024 using an online survey hosted on Google Forms, a secure web-based survey platform. The survey link was distributed via personalized email invitations disseminated through the official communication channels of the General Council of Nursing Colleges, the General Council of Medical Colleges, and national social work professional bodies in Spain. In addition, publicly accessible links were shared through institutional newsletters and professional mailing lists.

Participation was voluntary and anonymous. Professionals were invited once, and 2 to 3 reminder emails were sent at weekly intervals, depending on the response rate, to maximize participation during the data collection period. No financial incentives were provided to participants. Responses were reviewed for potential duplicate entries based on timestamp patterns. All submitted questionnaires were retained in the dataset. Descriptive analyses were based on all available data, whereas multivariable analyses were conducted using complete-case analysis, excluding participants with missing values for variables included in each model.

### Data Analysis

#### Quantitative Analyses

Descriptive statistics were calculated for all study variables, including means, SDs, medians, ranges, and frequencies, as appropriate. Item-level descriptive statistics were computed for the DigiHealthCom and the DigiComInf, and subscale and total scores were calculated as the mean of their respective items, with higher scores indicating higher levels of digital health competence or educational and organizational factors associated with the development of digital competence, respectively.

The internal consistency of the DigiHealthCom and the DigiComInf was assessed using Cronbach α and McDonald ω, with values of 0.70 or higher indicative of acceptable reliability [33].

Group differences in DigiHealthCom and DigiComInf total and domain scores by gender (women vs men) were examined using Welch independent-samples *t* tests, as the groups differed in size. Effect sizes were reported as Cohen *d*. Differences in DigiHealthCom and DigiComInf scores by highest educational achievement were analyzed using 1-way ANOVA, and effect sizes were reported as eta squared (η²). Associations between age and DigiHealthCom and DigiComInf scores were examined using Pearson correlation coefficients. All statistical tests were 2-tailed, and a *P* value <.05 was considered statistically significant. Where appropriate, 95% CIs are reported to complement point estimates and *P* values.

Confirmatory factor analyses (CFA) were conducted using maximum likelihood estimation, following the analytical approach described by Jarva et al [24]. Maximum likelihood estimation was selected to ensure methodological consistency with the original validation study and because the instruments use 4-point Likert scales, for which this estimator has been shown to provide reliable parameter estimates under appropriate sample size conditions. Model fit was evaluated using a combination of absolute and incremental goodness-of-fit indices [34]. The chi-square statistic was reported, acknowledging its sensitivity to sample size and model complexity. Model fit was primarily assessed using the root-mean-square error of approximation (RMSEA) and the standardized root-mean-square residual (SRMR), with values below 0.08 considered acceptable. Incremental fit was evaluated using the comparative fit index (CFI), Tucker-Lewis index (TLI), and incremental fit index (IFI), with values of 0.90 or higher indicating acceptable model fit.

#### Qualitative Analysis

AIQ responses were examined using inductive qualitative content analysis with a complementary summative element [35,36]. Responses were read repeatedly to gain familiarity with the data. Meaning units were identified and coded, and codes were progressively organized into subcategories and subsequently synthesized into broader themes. Two researchers (SJ-G and MFV-M) independently coded the dataset and developed a shared codebook through consensus discussions. Any discrepancies were addressed by returning to the original text until consensus was achieved. An audit trail, including analytic memos, codebook versions, and methodological decisions, was maintained throughout the process. For reporting purposes, code labels and theme names were translated from Spanish into English by agreement, while the original Spanish dataset and codebook were preserved. A word cloud and a Sankey diagram were used to complement the analytical narrative and enhance transparency.

#### Quantitating AI Attitudes and Association Testing

To address O4, AIQ responses were quantified into a mutually exclusive 4-category attitude variable (positive or opportunity-focused, negative or risk-focused, ambivalent or mixed, and uncertain or unclear). Two researchers (SJ-G and MFV-M) independently assigned each response to one category and resolved discrepancies by consensus. Intercoder agreement before consensus was assessed using Cohen κ. An audit trail, including analytic memos and documented coding decisions, was maintained to support dependability and confirmability. For reporting purposes, code labels and theme names were translated from Spanish into English by consensus, while the original Spanish dataset and codebook were retained.

As the sex category “prefer not to disclose” comprised a single case, it was excluded from analyses. Associations between AI attitude and sex were examined using chi-square tests and Cramér V. Differences in DigiHealthCom across attitude categories were examined using 1-way ANOVA, supplemented by Welch ANOVA and Kruskal-Wallis tests as robustness checks given unequal group sizes. Tukey Honestly Significant Difference was used for post hoc comparisons. A multinomial logistic regression model was fitted with negative attitude as the reference category, including DigiHealthCom total score, age, and sex as predictors; results were reported as odds ratios with 95% CIs. The multinomial model used listwise deletion (complete cases).

#### Software

Statistical analyses were performed using IBM SPSS Statistics (version 28) [37]. Confirmatory factor analyses were conducted using JASP (version 0.95.4) [38]. Qualitative data management and visualizations were carried out using ATLAS.ti (version 25) [39].

### Ethical Considerations

Permission to use the DigiHealthCom and DigiComInf was obtained from the original authors. Ethical approval for this study was granted by the Research Ethics Committee of the University of Alicante, Spain (UA-2023-04-03). Participation was voluntary, and informed consent was obtained electronically before completing the survey. Participants were informed of their right to withdraw from this study at any time without consequences.

This study was conducted in accordance with the principles of the Declaration of Helsinki [40], ensuring respect for participants’ autonomy, privacy, and well-being. Data were collected through an anonymous online questionnaire, and researchers had no direct contact with participants at any stage of this study. All data were anonymized and handled confidentially in accordance with secure data management procedures. This study also complied with the EU’s General Data Protection Regulation [41], ensuring the responsible processing and protection of personal data.

## Results

### Participants’ Demographic and Professional Characteristics

A total of 229 health care professionals completed the survey (Table 1). The sample was predominantly female, with women representing nearly three-quarters of respondents, while approximately one-quarter were men. Participants were, on average, middle-aged, with a broad age range from early adulthood to late working age. Most respondents reported high levels of educational attainment. The majority held at least a bachelor’s degree, and a substantial proportion had completed master’s or doctoral studies, indicating a highly educated professional sample.

In terms of professional background, nurses were the largest group, accounting for more than half of the sample, followed by public health nurses. Other professions were represented in smaller proportions, reflecting a multidisciplinary workforce.

**Table 1.** Demographic, professional, and work-related characteristics of health care professionals (N=229). Values are presented as n (%) unless otherwise indicated. Percentages are calculated using the total sample (N=229) and may not sum to 100.0 due to rounding.

Characteristic	Participants
Sex, n (%)	
Female	169 (73.8)
Male	59 (25.8)
Prefer not to disclose	1 (0.4)
Age, years	
Mean (SD)	45.9 (10.0)
Range	21‐64
Higher education at the highest level, n (%)	
Doctoral degree	17 (7.4)
Master’s degree	100 (43.7)
Bachelor’s degree	109 (47.6)
Other	3 (1.3)
Profession, n (%)	
Registered nurse	123 (53.7)
Public health nurse	31 (13.5)
Physician	18 (7.9)
Physiotherapist	19 (8.3)
Midwife	8 (3.5)
Social worker	6 (2.6)
Radiographer	2 (0.9)
Occupational therapist	1 (0.4)
Other	17 (7.4)
Clinical working environment/unit, n (%)	
Inpatient ward (including hospital ward, emergency ward, intensive care unit, operating theater, delivery room)	88 (38.4)
Outpatient services	47 (20.5)
Emergency services (also on call department)	25 (10.9)
Administration and research	14 (6.1)
Home care/rehabilitation or hospital-at-home	10 (4.4)
Social care (including integrated social work, disability service, family service, older adult service)	7 (3)
Other	38 (16.6)
Frequency of patient/customer contact, n (%)	
Daily (≥5 days/week)	143 (62.4)
Weekly (1‐4 days/week)	36 (15.7)
Monthly (several times/month)	10 (4.4)
Less often (a few times over several months)	6 (2.6)
Not currently working with patients/clients	34 (14.8)

In the clinical context, more than one-third of participants worked in inpatient care settings, while outpatient and emergency services were also well represented. Smaller proportions were employed in administration, research, home-based care, or social care services, highlighting the diversity of work environments included in this study. Most respondents reported frequent contact with patients or clients, with nearly two-thirds indicating daily contact. However, a notable minority reported limited or no contact with current patients or clients at the time of data collection.

### Psychometric Properties of the DigiHealthCom and DigiComInf

#### Content Validity

Content validity indices indicated excellent expert agreement for both instruments (Checklist 1). For DigiHealthCom, item-level content validity indices (I-CVI) were 0.96 for both relevance and clarity, and scale-level indices (S-CVI/Ave) were 0.969 (relevance) and 0.964 (clarity). For DigiComInf, I-CVI values were 0.98 for both relevance and clarity, and S-CVI/Ave values were 0.986 (relevance) and 0.968 (clarity).

#### CFA of DigiHealthCom

A CFA was conducted to test the hypothesized 5-factor structure of the DigiHealthCom (Checklist 1). The chi-square test was statistically significant (*χ*²_809_=1256.21, *P*<.001), which is common in large samples. IFIs indicated good model fit, with CFI 0.95, TLI 0.95, IFI 0.95, and Relative Noncentrality Index 0.95. Absolute and residual-based indices also supported a satisfactory fit: RMSEA 0.049 (90% CI 0.044‐0.054) and SRMR 0.059. Overall, the pattern of indices indicates a close approximate fit and supports the structural validity of the 5-factor model in this sample.

#### CFA of DigiComInf

The 3-factor model of DigiComInf was also evaluated via CFA (Multimedia Appendix 1). The chi-square test was significant (*χ*²_87_=227.003, *P*<.001). IFIs were satisfactory (CFI 0.95, TLI 0.94, IFI 0.95, and Relative Noncentrality Index 0.95). The SRMR indicated good residual fit (0.050), whereas the RMSEA was 0.084 (90% CI 0.071‐0.097), suggesting a borderline model fit. Although this value slightly exceeds the conventional 0.08 threshold, the strong IFIs and low SRMR support an overall acceptable model fit. Nevertheless, the RMSEA indicates that the structural validity of this instrument should be interpreted with appropriate caution and may benefit from further refinement in future validation studies.

#### Reliability

Both instruments demonstrated excellent internal consistency (Checklist 1). For DigiHealthCom, overall McDonald ω was 0.97, and Cronbach α was 0.96. Domain-level coefficients ranged from 0.87 to 0.98 (ω) and 0.86 to 0.96 (α). For DigiComInf, overall ω was 0.95, and α was 0.93, with subscale coefficients ranging from 0.85 to 0.94 for both indices. The results indicated high reliability at both the instrument and domain levels.

### Levels of Digital Health Competence Among Health Care Professionals (DigiHealthCom)

The DigiHealthCom scale showed excellent internal consistency at both total and domain levels (Table 2). Digital health competence was moderate overall, with higher scores observed in ICT-related competences and lower scores in domains related to the evaluation and implementation of digital solutions. Item-level results are in Multimedia Appendix 2.

No statistically significant differences were observed between women and men in the overall competence or across domains, with negligible effect sizes (Table 2). Age was not significantly associated with DigiHealthCom scores, indicating no linear relationship between age and digital health competence in this sample.

In contrast, the highest educational achievement was associated with slightly higher competence levels. This association was statistically significant but small in magnitude, suggesting limited practical impact.

**Table 2.** Digital health competence (DigiHealthCom): descriptive statistics and comparisons by gender. Group differences were tested using Welch *t* test. Effect sizes are reported as Cohen *d*.

	Total, mean (SD)	Women (n=169), mean (SD)	Men (n=59), mean (SD)	*t* test (Welch)	*P* value	Cohen *d*
DigiHealthCom total score (α=.96)	3.01 (0.51)	3.02 (0.47)	3.00 (0.62)	0.2	.84	0.03
Human-centered remote counseling competence (α=.96)	2.92 (0.65)	2.94 (0.59)	2.89 (0.79)	0.4	.69	0.06
Digital solutions as part of work (α=.95)	3.15 (0.68)	3.13 (0.65)	3.20 (0.77)	−0.6	.55	−0.09
Information and communication technology competence (α=.86)	3.34 (0.63)	3.39 (0.56)	3.20 (0.79)	1.69	.99	0.28
Competence in using and evaluating digital solutions (α=.92)	2.83 (0.63)	2.84 (0.58)	2.84 (0.77)	−0.04	.97	−0.01
Ethical competence related to digital solutions (α=.94)	2.98 (0.70)	2.97 (0.63)	3.04 (0.85)	−0.53	.59	−0.09

### Educational and Organizational Factors Associated With the Development of Digital Competence (DigiComInf)

The DigiComInf scale showed excellent internal consistency at both total and domain levels (Table 3). Organizational and educational factors supporting digital competence were moderate, with relatively stronger perceptions of collegial influence and weaker evaluations of structured organizational practices. Item-level results are presented in Multimedia Appendix 3.

No statistically significant differences were observed between women and men in overall scores or across domains, with negligible effect sizes (Table 3). Age was not associated with DigiComInf scores, indicating no linear relationship between age and perceived organizational or educational support.

Similarly, no significant differences in perceptions of organizational support for digital competence development were found, with trivial effect sizes, indicating consistency across subgroups.

**Table 3.** Organizational and educational factors (DigiComInf): descriptive statistics and comparisons by gender. Group differences were tested using Welch *t* test. Effect sizes are reported as Cohen *d*.

	Total, mean (SD)	Women (n=169), mean (SD)	Men (n=59), mean (SD)	*t* test (Welch)	*P* value	Cohen *d*
DigiComInf total score (α=.92)	2.51 (0.57)	2.52 (0.56)	2.47 (0.59)	0.49	.63	0.08
Support from management (α=.94)	2.59 (0.76)	2.61 (0.75)	2.55 (0.79)	0.56	.58	0.09
Organizational practices as part of digital competence development (α=.92)	2.21 (0.76)	2.20 (0.76)	2.19 (0.77)	0.13	.89	0.02
Colleagues’ adoption and influence (α=.85)	2.64 (0.59)	2.65 (0.58)	2.61 (0.62)	0.45	.66	0.07

### Perceptions of AI in the Workplace

Qualitative content analysis identified several interrelated themes and subthemes reflecting health care professionals’ expectations, concerns, and perceived implications of AI in the workplace and across health systems (Table 4). Their perceptions combined anticipated benefits with important ethical, professional, and organizational concerns. The most prominent theme, “adaptation and challenges,” highlighted the need to adjust to AI-enabled workflows, including training requirements, role changes, uncertainty, and concerns about depersonalization and unequal adoption.

The theme “patient care impact” reflected expectations of improved care quality, early detection, and personalized care, alongside awareness of potential effects on the patient-provider relationship. Similarly, “efficiency and workflow” highlighted anticipated gains in process optimization, reduced administrative burden, and support for evidence-based practice.

At the same time, participants emphasized “ethics and regulation” and “human oversight,” underscoring the need for clear governance, data protection, accountability, and the preservation of professional responsibility, with AI viewed primarily as a decision-support tool rather than a replacement for clinicians. Additional themes related to “resource constraints,” “sustainability,” and “accessibility” pointed to AI as a potential response to workforce shortages and to enhance system efficiency and access to care.

**Figure 2.**
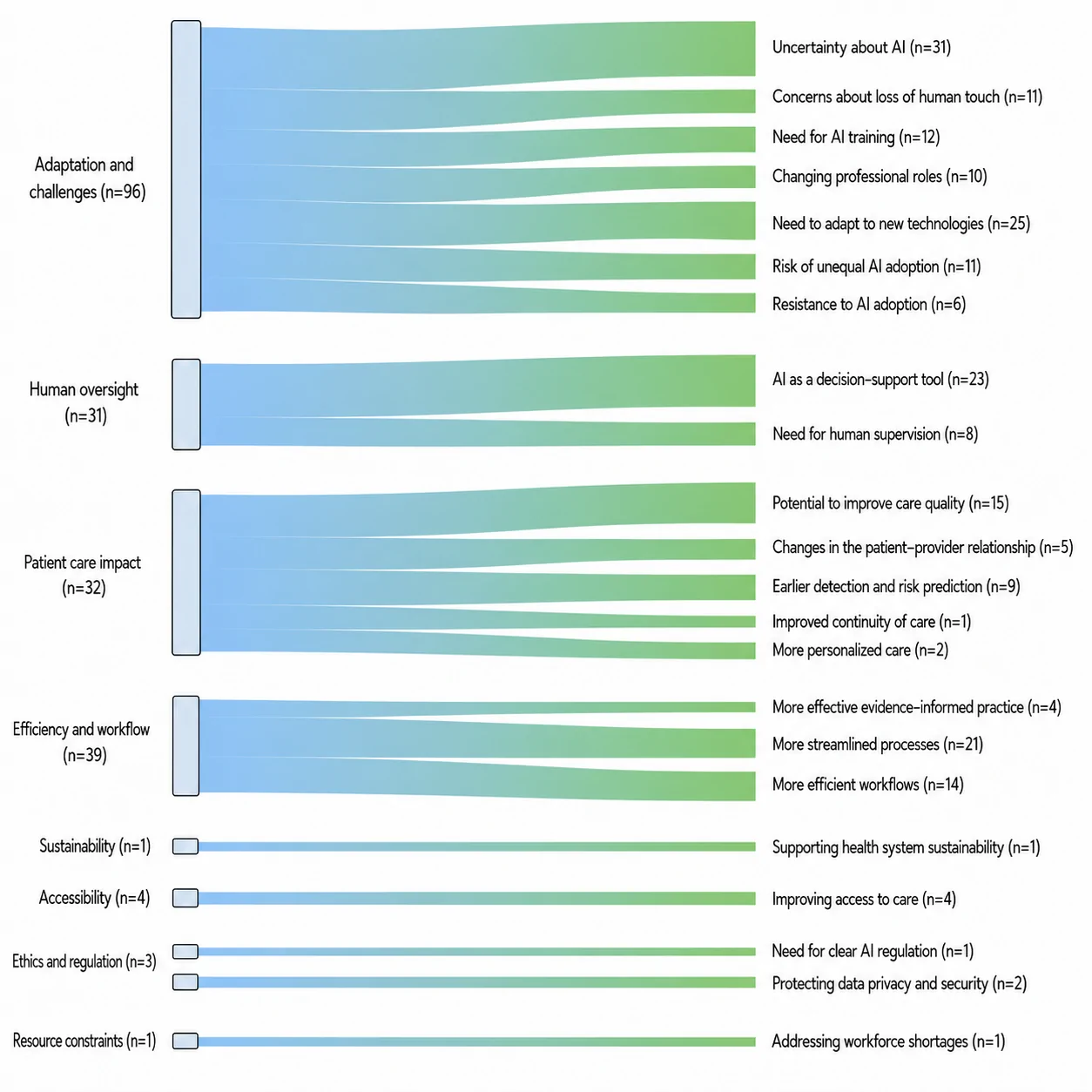
Sankey diagram of themes and subthemes derived from health care professionals’ perceptions of AI in clinical practice.

These themes together indicate a pattern of conditional acceptance of AI, shaped by perceived benefits but moderated by ethical, organizational, and professional considerations. Visual analyses supported these findings: the word cloud (Multimedia Appendix 4) emphasized central concepts such as patient care, workload, and professional roles, while the Sankey diagram (Figure 2) illustrated the prominence of adaptation-related challenges and their connections with training, uncertainty, and workflow changes.

**Table 4.** Themes and subthemes from qualitative analysis of health care professionals’ perceptions of AI (N=229).

Theme	Subthemes	Brief definition	Illustrative quotation
Adaptation and challenges	Adapting to new technologies Changing roles and job displacement Training and learning challenges Loss of human touch/depersonalization Unequal adoption (digital divide) High expectations vs reality Uncertainty and apprehension Resistance to AI adoption	Perceived need to adjust to AI-enabled workflows, including competence development and managing uncertainty and unintended consequences.	“I feel uncertain; I am not sufficiently informed about AI [...] it cannot replace direct contact with the patient to build empathy.” (R38)
Patient care impact	Improved care quality and outcomes Impact on the patient–provider relationship Early detection and risk prediction Personalized and tailored care Continuity of care	Expected effects of AI on clinical decision-making and care delivery, ranging from quality improvement to changes in the therapeutic relationship.	“I like the idea of using it predictively [...] to anticipate patient deterioration. It could make work much easier.” (R14)
Efficiency and workflow	Streamlined processes Workflow optimization Improved effectiveness (evidence-based)	Anticipated benefits of AI for reducing administrative burden, standardizing tasks, and supporting evidence-informed practice.	“It will help us reduce hours spent on administrative tasks, so we can work more with and for the patient.” (R154)
Ethics and regulation	Need for regulation Data privacy and security	Concerns about governance, accountability, privacy, and safe implementation of AI in health care.	“I am concerned about the lack of regulation in the use of AI.” (R55)
Human oversight	Need for human supervision AI as a decision-support tool	Emphasis on keeping clinical responsibility with professionals and using AI as support rather than replacement.	“It will be helpful, with appropriate supervision.” (R82)
Resource constraints	Workforce shortages (aging population)	AI is a potential response to limited human resources and increasing care demands.	“It will be the future of my profession because there are fewer caregivers and an aging population.” (R89)
Sustainability	Ensure health care system sustainability	Perceived role of AI in supporting system-level sustainability by improving efficiency and resource allocation.	“It is essential to ensure the sustainability of the health system.” (R110)
Accessibility	Remote access to care	Potential for AI-enabled tools to improve access, availability, and service reach for users.	“It would improve availability and accessibility for users.” (R48)

### Association Between Digital Health Competence and AI Attitudes

AI attitudes were predominantly positive, followed by uncertain or unclear, ambivalent, and negative orientations. Intercoder agreement for attitude classification was excellent (κ=0.949). No significant differences in AI attitudes were observed by sex or age, indicating that attitudinal variation was not explained by these demographic factors (Multimedia Appendices 5 and 6).

In contrast, digital health competence differed significantly across attitude categories (Table 5). Participants with a positive attitude toward AI reported higher competence levels than those with negative or uncertain attitudes, reflecting a moderate effect size.

Multinomial logistic regression analysis confirmed this pattern: higher digital health competence was independently associated with greater odds of expressing a positive attitude toward AI than a negative one, after adjusting for age and sex. No significant associations were observed for ambivalent or uncertain attitudes (Multimedia Appendix 7).

These quantitative findings are consistent with the qualitative results, which indicated a pattern of conditional acceptance of AI, shaped by perceived benefits but moderated by concerns about training, governance, and professional responsibility. The findings suggest that participants reporting higher levels of digital health competence were more likely to express positive and confident attitudes toward AI, whereas lower competence was associated with uncertainty or more cautious attitudes.

**Table 5.** Digital health competence (total score) across AI attitude categories (N=228; female n=168; male n=6). Group differences were tested using 1-way ANOVA with a Welch ANOVA robustness check (Welch *F*_3,62.312_=5.876, *P*=.001) and a Kruskal-Wallis test (H_3_=18.482, *P*<.001). Post hoc comparisons used Tukey HSD^a^; significant contrasts were positive and negative (*P*=.002) and positive and uncertain (*P*=.02).

Variable	Total (N=228)		Positive (n=134)		Negative (n=25)		Ambivalent (n=28)		Uncertain (n=41)		*F* (*df*=3,224)	*P* value	η²
	Mean (SD)	95% CI	Mean (SD)	95% CI	Mean (SD)	95% CI	Mean (SD)	95% CI	Mean (SD)	95% CI			
Digi Health Com total score	3.01 (0.51)	2.94‐3.08	3.11 (0.47)	3.03‐3.20	2.75 (0.51)	2.54‐2.97	2.96 (0.48)	2.78‐3.15	2.86 (0.56)	2.69‐3.04	5.424	<.001	0.068

a HSD: Honestly Significant Difference.

## Discussion

### Principal Findings

This convergent mixed methods study examined digital health competence, associated factors, and attitudes toward AI among health care professionals in Spain. Three principal findings emerged.

First, digital health competence was moderate overall, with stronger performance in ICT-related domains and weaker performance in evaluative and implementation competencies. Second, perceived organizational support for competence development was limited. Third, AI attitudes were predominantly positive but conditional, reflecting both expected benefits and concerns related to training, governance, and professional responsibility. Importantly, quantitative and qualitative findings converged to show that higher digital health competence was associated with more positive attitudes toward AI, suggesting that digital health competence may represent one of several factors associated with professionals’ attitudes toward AI.

### Digital Health Competence Profile and Organizational Context

The overall competence profile observed in this Spanish sample aligns with prior international evidence. Participants reported relatively strong ICT competence but lower scores in domains related to evaluation, implementation, and the critical appraisal of digital solutions. This pattern is consistent with systematic reviews indicating that health care professionals often demonstrate confidence in operational digital skills while reporting gaps in higher-order competencies such as critical evaluation and strategic integration [7,8].

Cross-national analyses using DigiHealthCom have similarly identified heterogeneity across domains and persistent weaknesses in advanced competence areas [27]. The confirmatory factor analyses in our study supported the structural validity of both DigiHealthCom and DigiComInf, reinforcing the robustness of the competence construct across contexts [21,24,25].

Associated factors of digital competence were perceived as only moderately developed, with structured planning and training consistently scoring lowest. This pattern aligns with prior research emphasizing the central role of managerial support, institutional practices, and formal training pathways in sustaining competence development [24,27]. The lack of significant associations with age and sex further suggests that variation in digital competence is more likely driven by structural and organizational factors than by individual demographic characteristics.

### AI Attitudes: Conditional Optimism

Qualitative findings revealed a pattern of conditional optimism toward AI. Most participants anticipated improvements in efficiency, decision support, and quality of care. However, these expectations were consistently linked to potential contributors such as adequate training, human oversight, regulatory clarity, and preservation of professional responsibility and patient-centered care.

Exclusively negative attitudes were uncommon. Rather, ambivalence reflected the coexistence of perceived opportunities and ethical or organizational concerns. This configuration aligns with prior studies indicating that health care professionals’ acceptance of AI is influenced by trust, perceived reliability, governance, and clear professional roles [4,11,12,23]. It is also consistent with empirical evidence showing that health care professionals’ perceptions of AI are generally favorable but nuanced, combining expectations of improved efficiency and clinical support with concerns about safety, accountability, and implementation context [42,43].

Participants’ emphasis on accountability and oversight resonates with the evolving regulatory framework under the EU Artificial Intelligence Act [29], which explicitly requires human oversight and adequate AI literacy in high-risk health care applications [44,45]. Although regulatory knowledge was not directly assessed, these findings suggest that professionals’ concerns reflect issues addressed in the current European regulatory framework.

### Digital Health Competence and Attitudes Toward AI

The central analytical contribution of this study lies in demonstrating an association between digital health competence and AI attitudes using validated instruments and multivariable modeling. Higher digital health competence was independently associated with a significantly greater likelihood of expressing a positive AI orientation, even after adjusting for age and sex. Although technology acceptance was not assessed directly, the observed association could be interpreted considering technology adoption frameworks such as TAM and UTAUT, which propose that perceived capability and facilitating conditions may influence engagement with new technologies [17,19]. In the present study, these frameworks were used to provide a theoretical context for interpreting the findings rather than to evaluate technology acceptance itself. Rather than representing a component of technology acceptance, digital health competence can be viewed as a complementary construct associated with, but conceptually distinct from, technology acceptance. This interpretation is complemented by the qualitative findings, in which participants consistently emphasized the importance of training, critical appraisal, and human oversight when discussing AI in clinical practice. Consistent with the exploratory nature of a single open-ended survey question and previous research, these qualitative findings primarily contextualize and support the quantitative results rather than providing novel conceptual insights.

Beyond technical proficiency, health care professionals reporting higher digital health competence may also perceive themselves as better able to interpret probabilistic outputs, recognize algorithmic limitations, evaluate data provenance, and integrate AI recommendations into clinical reasoning. These capacities may be related to greater perceived usefulness and trust, two factors consistently associated with AI adoption in health care. Conversely, lower perceived competence may also be associated with greater perceived risk, uncertainty, and concerns regarding liability or loss of autonomy, themes frequently highlighted in qualitative studies of AI implementation [13,23]. However, these potential mechanisms were not directly examined in the present study and may also reflect broader individual or organizational factors.

These findings indicate that digital health competence is associated with attitudes toward AI. However, the mechanisms underlying this relationship remain uncertain and are likely to involve broader individual, organizational, and contextual factors that were not examined in this study.

### Competence Gaps in the Regulatory Context

Participants reported a lack of structured institutional strategies for developing digital competence, with training largely dependent on individual initiative. This gap is consequential in a regulatory environment that requires not only formal compliance but also demonstrable capacity for oversight, accountability, and risk management.

The lowest competence levels were observed in domains related to evaluating and implementing digital solutions, precisely those required for meaningful human oversight under the EU Artificial Intelligence Act [29]. At the national level, Spain’s AI strategy for the National Health System (2025) similarly emphasizes training and capacity-building. The present findings provide empirical support for prioritizing advanced competence domains within such policy frameworks.

### Implications for Practice and Policy

The association between digital health competence and AI attitudes indicates that competence development may support more constructive engagement with AI in the workplace. Training should therefore prioritize advanced competencies, including the critical evaluation of digital tools, the interpretation of algorithmic outputs, their integration into clinical decision-making, and ethical-legal aspects such as accountability and data protection, in line with emerging European regulatory requirements [29,45].

At the organizational level, individual training must be complemented by structured strategies, protected time, interdisciplinary collaboration, and clear governance mechanisms to ensure safe and accountable implementation. Consistent with previous research [24,27], strengthening organizational support for digital health appears essential to align workforce preparedness with regulatory expectations and enable sustainable AI integration in health care.

### Strengths and Limitations

This study combined psychometrically supported instruments with confirmatory factor analysis (CFA) and a convergent mixed methods design, allowing both structural assessment and qualitative contextualization of health care professionals’ digital health competence and attitudes toward AI. The intercoder agreement achieved during AI attitude classification further supports the consistency of the qualitative analysis.

However, several limitations should be acknowledged. The use of a convenience sample may have introduced self-selection bias, and the predominance of nurses, together with the inability to calculate an overall response rate, limits the representativeness of the sample. As this study was conducted exclusively in one country, the findings should be generalized cautiously to other health care settings. Digital health competence was assessed through self-report and may reflect perceived rather than actual competence. Consequently, the observed association with AI attitudes may partly reflect generalized technological confidence rather than actual differences in digital health competence. In addition, the qualitative findings were derived from a single open-ended survey question, providing useful contextual information but less depth than interviews or focus groups. Finally, the multinomial regression model was adjusted only for age and sex, and other professional and organizational factors may also have influenced attitudes toward AI. Together with the cross-sectional design, this limits causal interpretation of the observed associations. Future longitudinal studies incorporating a broader range of explanatory variables are needed to clarify these relationships.

### Conclusions

Digital health competence was positively associated with health care professionals’ attitudes toward AI in this national sample. Although the cross-sectional design precludes causal inference, the findings suggest that professionals reporting higher levels of digital health competence are more likely to express positive attitudes toward AI. Additionally, lower scores in evaluative, implementation, and ethical competence domains, together with limited organizational support, highlight areas that may benefit from targeted educational and organizational strategies.

Future longitudinal and intervention studies are needed to clarify the direction of this association and to determine whether changes in digital health competence are accompanied by changes in attitudes toward AI and its implementation in health care.

## Supplementary material

10.2196/96170 Multimedia Appendix 1 Psychometric properties of DigiHealthCom (digital health competence instrument) and DigiComInf (aspects associated with digital health competence instrument; N=229).

10.2196/96170 Multimedia Appendix 2 Item-level descriptive statistics for the DigiHealthCom (digital health competence instrument).

10.2196/96170 Multimedia Appendix 3 Item-level descriptive statistics for the DigiComInf (aspects associated with digital health competence instrument).

10.2196/96170 Multimedia Appendix 4 Word cloud of health care professionals' perceptions of AI in clinical practice.

10.2196/96170 Multimedia Appendix 5 Distribution of AI attitude categories by sex.

10.2196/96170 Multimedia Appendix 6 Age distribution across AI attitude categories.

10.2196/96170 Multimedia Appendix 7 Multinomial logistic regression model predicting AI attitudes.

10.2196/96170 Checklist 1 STROBE Checklist of items that should be included in reports of cross-sectional studies.

## Abbreviations

AIQ: AI question
CFA: confirmatory factor analysis
CFI: comparative fit index
DigiComInf: aspects associated with digital health competence instrument
DigiHealthCom: digital health competence instrument
EU: European Union
I-CVI: item-content validity index
ICT: information and communication technology
IFI: incremental fit index
RMSEA: root-mean-square error of approximation
RO: research objective
S-CVI/Ave: scale-level content validity index and average calculation method
SRMR: standardized root-mean-square residual
STROBE: Strengthening the Reporting of Observational Studies in Epidemiology
TAM: technology acceptance model
TLI: Tucker-Lewis index
UTAUT: unified theory of acceptance and use of technology
