# Supplementary material for: Digital Health Competence and Attitudes Toward AI Among Health Care Professionals: Convergent Mixed Methods Study
Source: J Med Internet Res. 2026 Aug 24;28:e96170. doi: 10.2196/96170 (PMC13503649; doi:10.2196/96170)
Supplement: Supplementary file 1 [file jmir-v28-e96170-s001.docx]

| **Property** | **DigiHealthCom** | **DigiComInf** |
| --- | --- | --- |
| **Content validity** |  |  |
| I-CVI (relevance) | 0.96 | 0.98 |
| I-CVI (clarity) | 0.96 | 0.98 |
| S-CVI/Ave (relevance) | 0.969 | 0.986 |
| S-CVI/Ave (clarity) | 0.964 | 0.968 |
| **Model fit (CFA)** |  |  |
| Chi-square statistics- χ² (df; p) | 1256.21 (809; <0.001) | 227.003 (87; <0.001) |
| Comparative Fit Index (CFI) | 0.95 | 0.95 |
| Trucker-Lewis Index (TLI) | 0.95 | 0.94 |
| Bollen’s Incremental Fit Index (IFI) | 0.95 | 0.95 |
| Relative Noncentrally Index (RNI) | 0.95 | 0.95 |
| RMSEA (90% CI bound) | 0.049 (0.044-0.054) | 0.084 (0.071–0.097) |
| SRMR | 0.059 | 0.050 |
| **Reliability** |  |  |
| McDonald’s omega (ω) | 0.97 | 0.95 |
| Cronbach’s alpha (α) | 0.96 | 0.93 |
| ω (range) | 0.87–0.98 | 0.85–0.94 |
| α (range) | 0.86–0.96 | 0.85–0.94 |

Note. CVI = content validity index; I-CVI = item-level CVI; S-CVI/Ave = scale-level CVI (average method); CFA = confirmatory factor analysis; RMSEA = Root mean square error of approximation; SRMR = Standardised root mean square residual
