# Supplementary material for: Digital Health Competence and Attitudes Toward AI Among Health Care Professionals: Convergent Mixed Methods Study
Source: J Med Internet Res. 2026 Aug 24;28:e96170. doi: 10.2196/96170 (PMC13503649; doi:10.2196/96170)
Supplement: Supplementary file 2 [file jmir-v28-e96170-s002.docx]

| **Domain /** Item | **n** | **Mean** | **Median** | **SD** | **Min** | **Ma** | **Percentiles** | | |
| --- | --- | --- | --- | --- | --- | --- | --- | --- | --- |
|  |  |  |  |  |  |  | **25** | **50** | **75** |
| **I. Human-centred remote counselling competence** |  |  |  |  |  |  |  |  |  |
| 1. I can act in reciprocal (aiming towards respect and equality) interaction with the customer in remote counselling | 229 | 3.11 | 3 | 0.80 | 1 | 4 | 3 | 3 | 4 |
| 2. I can set goals together with the customer in remote counselling | 229 | 3.12 | 3 | 0.77 | 1 | 4 | 3 | 3 | 4 |
| 3. I can form a confidential relationship with the customer in remote counselling | 229 | 3.12 | 3 | 0.86 | 1 | 4 | 3 | 3 | 4 |
| 4. I can recognise the customer’s need for support and guidance in remote counselling | 229 | 2.84 | 3 | 0.83 | 1 | 4 | 2 | 3 | 3 |
| 5. I can motivate the customer into action/self-care in remote counselling | 229 | 2.93 | 3 | 0.82 | 1 | 4 | 3 | 3 | 3 |
| 6. I can take into consideration the special characteristics of online interaction (e.g. wording. addressing empathy) in remote counselling | 229 | 2.82 | 3 | 0.84 | 1 | 4 | 2 | 3 | 3 |
| 7. I can recognise when the customer’s service (e.g. care or guidance) can be delivered remotely | 229 | 3.04 | 3 | 0.77 | 1 | 4 | 3 | 3 | 4 |
| 8. I can guide the customer verbally in remote counselling (e.g. on the phone without video) | 229 | 2.95 | 3 | 0.73 | 1 | 4 | 3 | 3 | 3 |
| 9. I can guide the customer by utilising a video connection in remote counselling | 229 | 3.00 | 3 | 0.78 | 1 | 4 | 3 | 3 | 3 |
| 10. I can evaluate the customer’s situation (need for care or service) in remote counselling | 229 | 2.73 | 3 | 0.82 | 1 | 4 | 2 | 3 | 3 |
| 11. I can guide the customer in writing (e.g. chat service) in remote counselling | 229 | 2.72 | 3 | 0.85 | 1 | 4 | 2 | 3 | 3 |
| 12. I can evaluate whether customers receive equal service in remote counselling | 229 | 2.65 | 3 | 0.86 | 1 | 4 | 2 | 3 | 3 |
| 13. I can recognise the customer’s willingness to use digital solutions | 229 | 2.90 | 3 | 0.78 | 1 | 4 | 3 | 3 | 3 |
| 14. I can act professionally in remote counselling | 229 | 3.02 | 3 | 0.79 | 1 | 4 | 3 | 3 | 4 |
| 15. I can evaluate the customer’s digital readiness | 229 | 2.81 | 3 | 0.84 | 1 | 4 | 2 | 3 | 3 |
| 16. I can guide the customer to find reliable information (e.g. Ministry of Health. Regional Health Authorities. Patient School. Health Promotion and Prevention. etc.) | 229 | 3.00 | 3 | 0.78 | 1 | 4 | 3 | 3 | 4 |
| **II. Digital solutions as part of work** |  |  |  |  |  |  |  |  |  |
| 1. The transfer to digital services is a positive change | 229 | 3.15 | 3 | 0.76 | 1 | 4 | 3 | 3 | 4 |
| 2. Digital solutions should be used more in social and health services | 229 | 3.10 | 3 | 0.82 | 1 | 4 | 3 | 3 | 4 |
| 3. I am motivated to use digital solutions in my work | 229 | 3.25 | 3 | 0.78 | 1 | 4 | 3 | 3 | 4 |
| 4. I consider digital solutions as useful | 229 | 3.33 | 3 | 0.67 | 1 | 4 | 3 | 3 | 4 |
| 5. I am interested in learning about digital solutions in my work | 229 | 3.35 | 3 | 0.74 | 1 | 4 | 3 | 3 | 4 |
| 6. Digital solutions support my work | 229 | 3.31 | 3 | 0.73 | 1 | 4 | 3 | 3 | 4 |
| 7. Digital services are a good way to deliver social and health services (e.g. customer work. care. rehabilitation) | 229 | 3.09 | 3 | 0.82 | 1 | 4 | 3 | 3 | 4 |
| 8. Digital solutions are a natural part of my work | 229 | 2.91 | 3 | 0.91 | 1 | 4 | 2 | 3 | 4 |
| 9. Digital solutions do not slow down my work | 229 | 2.86 | 3 | 0.94 | 1 | 4 | 2 | 3 | 4 |
| **III. Information and communication technology (ICT) competence** |  |  |  |  |  |  |  |  |  |
| 1. I can use the most common computer programs and services (e.g. email. intranet) in my work | 229 | 3.53 | 4 | 0.72 | 1 | 4 | 3 | 4 | 4 |
| 2. I can use equipment based on information technology (e.g. computer) in my work | 229 | 3.54 | 4 | 0.70 | 1 | 4 | 3 | 4 | 4 |
| 3. I can ask for help in information technology issues (e.g. ICT support) | 229 | 3.21 | 3 | 0.80 | 1 | 4 | 3 | 3 | 4 |
| 4. I can use the patient/client information system in my work | 229 | 3.23 | 3 | 0.82 | 1 | 4 | 3 | 3 | 4 |
| 5. I can solve most common information technology challenges (e.g. login problems. display settings. printer settings) in my work | 229 | 3.16 | 3 | 0.85 | 1 | 4 | 3 | 3 | 4 |
| **IV. Competence in utilising and evaluating digital solutions** |  |  |  |  |  |  |  |  |  |
| 1. I can recognise what digital solutions are in social and health services | 229 | 2.85 | 3 | 0.76 | 1 | 4 | 2 | 3 | 3 |
| 2. I can recognise factors (e.g. resources. motivation) that influence the utilisation of digital solutions | 229 | 2.94 | 3 | 0.71 | 1 | 4 | 3 | 3 | 3 |
| 3. I can utilise digital solutions (e.g. smart devices. applications) in customer care/guidance | 229 | 2.93 | 3 | 0.76 | 1 | 4 | 2 | 3 | 3 |
| 4. I can utilise digital solutions creatively (e.g. usage according to different customer needs) in my work | 229 | 2.69 | 3 | 0.84 | 1 | 4 | 2 | 3 | 3 |
| 5. I can boldly experiment and implement digital solutions in my work | 229 | 2.64 | 3 | 0.85 | 1 | 4 | 2 | 3 | 3 |
| 6. I can explain digital social and health services (e.g. mobile applications of the Autonomous Health Agency) to customers | 229 | 2.70 | 3 | 0.82 | 1 | 4 | 2 | 3 | 3 |
| 7. I can use my professional skills when using digital solutions | 229 | 3.00 | 3 | 0.76 | 1 | 4 | 3 | 3 | 4 |
| 8. I can critically evaluate new digital solutions | 229 | 2.90 | 3 | 0.79 | 1 | 4 | 2 | 3 | 3 |
| **V. Ethical competence related to digital solutions** |  |  |  |  |  |  |  |  |  |
| 1. I can secure the customer’s privacy when using digital solutions | 229 | 2.98 | 3 | 0.77 | 1 | 4 | 3 | 3 | 3 |
| 2. I can ensure the secure processing of customer data | 229 | 2.99 | 3 | 0.76 | 1 | 4 | 3 | 3 | 3 |
| 3. I can acknowledge the customer’s autonomy when using digital solutions | 229 | 3.00 | 3 | 0.78 | 1 | 4 | 3 | 3 | 4 |
| 4. I can recognise the ethical aspects of digital solutions (e.g. freedom of choice. privacy. fairness) | 229 | 2.96 | 3 | 0.75 | 1 | 4 | 3 | 3 | 3 |
