# Supplementary material for: Digital Health Competence and Attitudes Toward AI Among Health Care Professionals: Convergent Mixed Methods Study
Source: J Med Internet Res. 2026 Aug 24;28:e96170. doi: 10.2196/96170 (PMC13503649; doi:10.2196/96170)
Supplement: Supplementary file 3 [file jmir-v28-e96170-s003.docx]

| **Domain /** Item | **n** | **Mean** | **Median** | **SD** | **Min** | **Ma** | **Percentiles** | | |
| --- | --- | --- | --- | --- | --- | --- | --- | --- | --- |
|  |  |  |  |  |  |  | **25** | **50** | **75** |
| **I. Support from management** |  |  |  |  |  |  |  |  |  |
| 1. My manager’s example supports the development of my digital competence | 229 | 2.57 | 3.0 | 0.87 | 1 | 4 | 2 | 3 | 3 |
| 2. My manager supports the implementation of digital solutions | 229 | 2.69 | 3.0 | 0.82 | 1 | 4 | 2 | 3 | 3 |
| 3. My manager gives feedback about the development of my digital competence | 229 | 2.36 | 2.0 | 0.87 | 1 | 4 | 2 | 2 | 3 |
| 4. My manager can lead the development of my digital competence (e.g. prediction of competence development, communication. clear guidance, support for renewal and participation) | 229 | 2.62 | 3.0 | 0.87 | 1 | 4 | 2 | 3 | 3 |
| 5. My manager supports my participation in continuing education to strengthen my digital competence | 229 | 2.66 | 3.0 | 0.89 | 1 | 4 | 2 | 3 | 3 |
| 6. Top management supports the uptake of digital solutions | 229 | 2.66 | 3.0 | 0.83 | 1 | 4 | 2 | 3 | 3 |
| **II. Organisational practices as part of digital competence development** |  |  |  |  |  |  |  |  |  |
| 1. Education about the digital solutions used at my work has been sufficient | 229 | 2.19 | 2.0 | 0.81 | 1 | 4 | 2 | 2 | 3 |
| 2. Digital competence development is planned in my unit according to individual needs | 229 | 2.14 | 2.0 | 0.82 | 1 | 4 | 2 | 2 | 3 |
| 3. The orientation for digital solutions is conducted systematically at my work unit | 229 | 2.21 | 2.0 | 0.86 | 1 | 4 | 2 | 2 | 3 |
| 4. My organisation’s practices support opportunities to develop my digital competence | 229 | 2.29 | 2.0 | 0.86 | 1 | 4 | 2 | 2 | 3 |
| **III. Colleagues’ adoption and influence** |  |  |  |  |  |  |  |  |  |
| 1. Colleagues are not reluctant to start using digital solutions at work | 229 | 2.62 | 3.0 | 0.77 | 1 | 4 | 2 | 3 | 3 |
| 2. The implementation of digital solutions has been perceived positively in my work community | 229 | 2.62 | 3.0 | 0.73 | 1 | 4 | 2 | 3 | 3 |
| 3. Colleagues are eager to develop their own work on digital solutions | 229 | 2.63 | 3.0 | 0.72 | 1 | 4 | 2 | 3 | 3 |
| 4. Colleagues do not have a negative influence on the development of my digital competence | 229 | 2.78 | 3.0 | 0.79 | 1 | 4 | 2 | 3 | 3 |
| 5. Colleagues in my work community have mainly a good level of digital competence | 229 | 2.55 | 3.0 | 0.76 | 1 | 4 | 2 | 3 | 3 |
