# Supplementary material for: Digital Health Competence and Attitudes Toward AI Among Health Care Professionals: Convergent Mixed Methods Study
Source: J Med Internet Res. 2026 Aug 24;28:e96170. doi: 10.2196/96170 (PMC13503649; doi:10.2196/96170)
Supplement: Supplementary file 4 [file jmir-v28-e96170-s005.docx]

|  | **Female** (n=168) | | **Male** (n=60) | | **Total** (n=228) | |
| --- | --- | --- | --- | --- | --- | --- |
| **AI attitude category** | n | % | n | % | n | % |
| Positive / opportunity-focused | 96 | 57.1 | 37 | 61.7 | 133 | 58.3 |
| Negative / risk-focused | 19 | 11.3 | 7 | 11.7 | 26 | 11.4 |
| Ambivalent / mixed | 22 | 13.1 | 6 | 10.0 | 28 | 12.3 |
| Uncertain / unclear | 31 | 18.5 | 10 | 16.7 | 41 | 18.0 |
| Column totals | 168 | 100.0 | 60 | 100.0 | 228 | 100.0 |

Note. Percentages are within sex. Pearson χ² (3, n=228) =0.583, p=0.900; Cramér’s V=0.051. No expected cell counts <5 (minimum expected count=6.84). One participant did not disclose sex and was excluded from sex-stratified analyses.
