# Supplementary material for: Digital Health Competence and Attitudes Toward AI Among Health Care Professionals: Convergent Mixed Methods Study
Source: J Med Internet Res. 2026 Aug 24;28:e96170. doi: 10.2196/96170 (PMC13503649; doi:10.2196/96170)
Supplement: Supplementary file 5 [file jmir-v28-e96170-s006.docx]

| **Variable** | **Positive** (n=134) | **Negative** (n=25) | **Ambivalent** (n=28) | **Uncertain** (n=41) |
| --- | --- | --- | --- | --- |
|  | **Mean (SD)** [95% CI] | **Mean (SD)** [95% CI] | **Mean (SD)** [95% CI] | **Mean (SD)** [95% CI] |
| **Age** (years) | 45.35 (9.46) [43.73–46.97] | 44.52 (10.72) [40.09–48.95] | 47.79 (10.85) [43.58–51.99] | 48.41 (8.39) [45.77–51.06] |

Note. Valid n=228; one missing age value in the negative attitude group. One-way ANOVA: F(3,224) = 1.579, p=0.195; Levene’s test p=0.227; Welch ANOVA p=0.181. Kruskal–Wallis test p=0.219.
