# Supplementary material for: Digital Health Competence and Attitudes Toward AI Among Health Care Professionals: Convergent Mixed Methods Study
Source: J Med Internet Res. 2026 Aug 24;28:e96170. doi: 10.2196/96170 (PMC13503649; doi:10.2196/96170)
Supplement: Supplementary file 6 [file jmir-v28-e96170-s007.docx]

| **Outcome (vs negative)** | **Predictor** | **OR** | **95% CI** | **p** |
| --- | --- | --- | --- | --- |
| **Positive** | DigiHealthCom total score | 4.47 | 1.81–11.06 | 0.001 |
|  | Age (years) | 1.01 | 0.97–1.06 | 0.610 |
|  | Sex (female vs male [reference]) | 0.88 | 0.32–2.39 | 0.795 |
| **Ambivalent** | DigiHealthCom total score | 2.28 | 0.74–6.97 | 0.148 |
|  | Age (years) | 1.04 | 0.98–1.10 | 0.203 |
|  | Sex (female vs male [reference]) | 1.28 | 0.35–4.63 | 0.706 |
| **Uncertain** | DigiHealthCom total score | 1.52 | 0.55–4.16 | 0.417 |
|  | Age (years) | 1.04 | 0.99–1.10 | 0.108 |
|  | Sex (female vs male [reference]) | 1.14 | 0.36–3.63 | 0.825 |

Note. Multinomial logistic regression model with negative AI attitude as the reference category; n=227 complete cases. Model fit: LR χ²(9) =21.853, p=0.009. Pseudo R²: Cox–Snell=0.092, Nagelkerke=0.103, McFadden=0.043. Predictors included DigiHealthCom total score, age, and sex (female vs male [reference]).
